# Vaccination and its impact on healthcare utilization in two groups of vaccinated and unvaccinated patients with COVID‐19: A cross‐sectional study in Iran between 2021 and 2022

**DOI:** 10.1002/hsr2.1914

**Published:** 2024-02-23

**Authors:** Erfan Kharazmi, Mohsen Bayati, Ali Majidpour Azad Shirazi

**Affiliations:** ^1^ Health Human Resources Research Center, School of Health Management and Information Sciences Shiraz University of Medical Sciences Shiraz Iran

**Keywords:** COVID‐19, Coronavirus, Utilization of Health Services, Vaccination

## Abstract

**Background and Aims:**

One of the main responsibilities of health systems impacted by the global Coronavirus disease 2019 (COVID‐19) pandemic, where the first case was discovered in Wuhan, China, in December 2019, is the provision of medical services. The current study looked into the impact of vaccination on the utilization of services provided to COVID‐19 patients.

**Methods:**

This study was conducted in Iran between 2021 and 2022, utilizing a cross‐sectional research design. The research team collected data on the utilization of provided services and the number of COVID‐19 vaccines administered to 1000 patients in Iran through a random sampling approach. The data were analyzed with statistical methods, including the mean difference test, and multiple linear regression.

**Results:**

Regression estimates show that after controlling for confounding variables like age, type of admission, and comorbidities, vaccination reduces the utilization of healthcare services in the general majority of services. The study's results reveal a fall in COVID‐19 patients' utilization of services, specifically in patients administered two or three doses of the vaccine. However, the reduction is not statistically significant. Regression models are in contrast to univariate analysis findings that vaccination increases the mean utilization of healthcare services for COVID‐19 patients in general. Comorbidities are a crucial factor in determining the utilization of diagnostic and treatment services for COVID‐19 patients.

**Conclusion:**

Full COVID‐19 vaccination and other implementations, including investing in public health, cooperating globally, and vaccinating high‐risk groups for future pandemics, are essential as a critical response to this pandemic as they reduce healthcare service utilization to alleviate the burden on healthcare systems and allocate resources more efficiently.

## 1 INTRODUCTION

On January 30, 2020, the World Health Organization (WHO) declared the new Coronavirus outbreak a global public health emergency. The disease was later named Coronavirus disease 2019 (COVID‐19). On March 11, 2020, it was classified as an epidemic. According to WHO reports, as of August 16, 2023, there would have been 769,806,130 confirmed cases of COVID‐19 worldwide, resulting in 6,955,497 fatalities.^1^

The COVID‐19 outbreak in Iran is a global crisis caused by the Coronavirus and its acute respiratory syndrome. Iran recorded its first case of COVID‐19 on February 19, 2020. Since then, there have been eight waves of epidemics, and as of August 16, 2023, there have been 7,613,468 cases of the disease with 146,321 fatalities, according to WHO.^2^

Since the first case in January 2020, the acute respiratory syndrome virus has been constantly evolving, resulting in various variants with mutations that alter receptor binding.^3^, ^4^ The Alpha variant of SARS‐CoV‐2, or B.1.1.7, was first identified in the United Kindom in September 2020 and rapidly spread.^5^ The Alpha variant, in 2021, was the most common strain of COVID‐19 in Denmark, Switzerland, and the United States. It is 56% more infectious than earlier strains.^6^, ^7^, ^8^ The Delta variant, B.1.617.2, was found in India in December 2020. It is 40%–60% more contagious than The Alpha variant.^9^ Notably, COVID‐19's impact on hospitalized patients has changed with new strains, treatments, and vaccines. But age and underlying health conditions remain risk factors for mortality across all strains.^10^

Vaccines are seen as a solution to the pandemic. COVID‐19 vaccines have efficacy rates from 65% to 95% against symptomatic cases.^4^ The evidence shows that injecting the vaccination before contracting COVID‐19 by creating immunity reduces the prevalence of symptomatic disease in patients.^11^ Full vaccination might be one of the finest COVID‐19 control measures, potentially preventing millions of deaths.^12^ The inactivated BBIBP‐CorV vaccine (Sinopharm), made in China, has a 79.34% effectiveness rate.^13^ New strains of SARS‐CoV‐2 may affect the effectiveness of COVID‐19 vaccines.^14^ Vaccines may be less effective against Delta variant. Pfizer's efficacy rate is 79% and AstraZeneca‐Oxford's is 60%.^14^, ^15^ Pfizer and AstraZeneca‐Oxford vaccines are less effective against the Delta variant with a single dose.^16^

The WHO reported that 13,499,865,692 vaccine doses had been administered globally until August 19, 2023.^1^ Boosters reduce severe illness and death for COVID patients without prior infection, but the effectiveness of vaccines may vary based on prior infections.^17^, ^18^ Another study found there is no correlation between previous SARS‐CoV‐2 infection and adverse events or hospitalization. However, those previously infected were less likely to visit the accident and emergency department after CoronaVac or Comirnaty vaccination.^19^ In a study using a model design, Reddy et al. asserted that prioritizing high‐risk groups and vaccinating at least 40% of the population in South Africa, saved more than nine million lives in prevalence and prevented more than 73,000 fatalities.^20^ Watson et al. found that if low‐income countries vaccinated 20% of their general population against COVID‐19, up to 45% of COVID‐19‐related deaths may be prevented. If this objective was adjusted to 40%, up to 111% of deaths may be prevented (based on the WHO target).^21^ Magazzino et al.'s machine learning analysis demonstrated a decrease in COVID‐19 fatalities as a result of successful vaccination campaigns and high public participation.^22^

The key to optimal COVID‐19 vaccination rates is through mathematical models, optimization algorithms, and prioritizing the vaccination of a larger number of older individuals first.^23^ This approach also involves vaccinating a portion of the younger population to reduce the risk of transmission between age groups.^24^ With regard to the optimal level of vaccination, this rate for COVID‐19 is around 80 doses per 100 people, but an intensive campaign can reduce this to 47 doses initially and increase it to around 90 doses as the pandemic wave intensifies.^25^

Global vaccine distribution is restricted by vaccine hesitancy, which stems from various factors including government, healthcare, population, lower education, unemployment, lack of vaccine information, concerns about side effects, and misinformation spread through social media.^26^, ^27^ Although some people may feel hesitant about getting the COVID‐19 vaccine because they are concerned about side effects such as fever, arm, and muscle pain, these reactions are often mild and do not last long.^28^ Reports of neurological side effects, including cerebrovascular and demyelinating disorders, are rare and temporary.^29^ Wealthy and developed nations tend to have higher vaccine hesitancy rates, which can reach up to 70%. Countries with stable governments and political systems tend to have higher vaccination rates. Monetary incentives may encourage hesitant individuals to get vaccinated.^30^ Also, Urban areas have higher rates of immunization due to disparities in vaccine distribution. This gap is worsened by factors such as maternal healthcare access, household wealth, number of children, and sources of information.^31^

We conducted this study to ascertain how the administration of the COVID‐19 vaccine affected the utilization of screening and treatment services that are given by health systems in light of Iran's higher ranking in the death rate and new COVID‐19 reported incidents (according to WHO reports on August 10, 2021, Iran had the highest daily incidence of new cases among other countries^32^) from the start of April 2021 (the start of the fifth wave of the epidemic) to the end of October 2021 (the end of the sixth wave of the epidemic) and the escalation of massive public vaccination in Iran from July 2021 with the administering of the first dose of the vaccine according to the WHO reports.^2^

## 2 METHODS

### 2.1 Sample and data

Between 2021 and 2022, we conducted a cross‐sectional study in Shiraz, Iran. Our sample consisted of 1000 participants who had previously tested positive for COVID‐19 via polymerase chain reaction (PCR), out of a pool of 3724 individuals. Of these participants, 349 were admitted to the hospital while 651 received outpatient services. We categorized the participants into two groups based on their vaccination status.

We acquired data from the Hospital Information System on patients' utilization of clinical and therapeutic services. The number of vaccine doses given and the date of injection for each participant were recorded in a form made by the researchers using the Integrated Health System. The information was obtained without exposing the patients' identities in the following ethical norms. Informed consent was not needed as all patients were anonymized and only aggregate data were used.

### 2.2 Measures of variables

There are three main categories of variables involved in this study. The number of vaccine doses given to the patient and whether or not the vaccine is injected are intervention factors. Dependent variables include the utilization of services in terms of visits and clinical consults, laboratory tests, medication, consumables, radiology, computerized tomography (CT) scans, surgeries, ultrasound, rehabilitation services, intensive care unit (ICU) hospitalization, non‐ICU hospitalization, dialysis, nutritional counseling, and blood gas test. Contextual variables include age, gender, type of insurance, residence, type of admission, and comorbidities including autoimmune disorders, cancers, diabetes, cardiovascular diseases, and respiratory diseases.

### 2.3 Models and data analysis procedure

After acquiring the data, we used Stata 17 to perform statistical analysis using various methods, including mean difference test and multiple linear regression. Performing mean difference tests, we assessed whether vaccination and the number of doses administered to patients are associated with a difference in the utilization of diagnostic and treatment services.

We conducted two multiple linear regression models: the first model examined the impact of vaccination on diagnostic and therapeutic service utilization while controlling for contextual variables, while the second model examined the impact of the number of doses given on diagnostic and treatment service utilization while controlling for contextual variables. We assessed regression coefficients at significant levels of 0.01, 0.05, and 0.10. Notably, the current study hypothesizes that COVID‐19 vaccination and booster doses reduce the utilization of diagnostic and treatment services for patients.

This study was approved by the ethics committee of Shiraz University of Medical Sciences under code IR.SUMS.NUMIMG.REC.1401.037.

## 3 RESULTS

According to the survey results, the average age of the participants was 51.48 ± 17.97. Out of the total 1000 participants, 1.6% suffered from autoimmune disorders, 0.9% suffered from cancer, 10% suffered from diabetes, 15.9% suffered from cardiac diseases, and 6.9% had respiratory disorders.

From 2021 to 2022, we gathered a sample of 1000 participants. Among them, 181 patients were not vaccinated before being referred to the hospital and receiving treatment and were therefore included in the group of unvaccinated patients. The remaining COVID‐19 patients were vaccinated at least a week before being referred to the hospital for treatment and were grouped as vaccinated patients. The vaccinated group was divided into two subgroups, where 274 received just one dose and 545 received two or three doses. Table 1 provides a comprehensive overview of the vaccination and demographic details of the 1000 participants.

**Table 1:** Participants' demographic and vaccination information in Iran between 2021 and 2022 (*n* = 1000).

Variable	Category	Number	Percentage
Gender	Female	519	51.90
	Male	481	48.10
Age	<20	17	1.7
	20–30	137	13.7
	31–40	124	12.4
	41–50	211	21.1
	51–60	203	20.3
	61–70	158	15.8
	>70	150	15
Type of Insurance	Social Security	491	49.10
	Health Service	361	36.10
	Armed Forces	60	6
	Other	34	3.40
	Non‐insured	54	5.40
Discharge	Recovery	861	86.10
	Death	64	6.40
	Transfer	5	0.50
	Voluntary Clearance	70	7
Location	Non‐province Center	30	3
	Province Center	970	97
Patients	Inpatient	349	34.90
	Outpatient	651	65.10
Vaccination	Non‐Vaccinated	819	81.90
	Vaccinated	181	18.10
Injected doses	Non‐Vaccinated	181	18.10
	1 Dose Administered	274	27.40
	2 or 3 Doses Administered	545	54.50

Using mean difference tests, vaccination in patients infected with COVID‐19 increases the overall mean utilization of diagnostic and treatment services. The utilization of visits, laboratory tests, consumables, medicines, ultrasounds, dialysis, radiology, blood gas test, echo, and nutritional counseling grow significantly following vaccination (Supporting Information S1: Table S1 in the appendices presents the results of mean difference test based on vaccination, and the normality distribution of each variable).

As follows, mean difference tests show that patients infected with COVID‐19 who received the second or third dose of vaccination tend to have higher utilization of diagnostic and therapeutic services compared to those who received only the first dose (Supporting Information S1: Table S2 in the appendices presents the results of mean difference test based on the number of vaccine doses administered.).

The first regression model explores the impact of vaccination on patient utilization of diagnostic and therapeutic services while accounting for contextual factors. The results are presented in Table 2. In this model, vaccination significantly reduces the use of CT scans, medication, rehabilitation services, hospitalization in the ICU, and hospitalization outside the ICU. Men undergo dialysis more frequently than women, and the use of medication and radiology increases with age.

**Table 2:** Variables affecting the utilization of services based on vaccination in Iran between 2021 and 2022.

	Length of non‐ICU stay	Length of ICU stay	Visits and Medical Counseling	Laboratory	Medicine	Ultrasound	Rehabilitation	Dialysis	Radiology	CT scan	Arterial Blood Gas test	Echo	Nutrition Advice	Surgery	Medical Supplies
Vaccination	−0.206	−0.160	0.007	−0.061	−0.337	0.001	−0.018	0.015	0.001	−0.025	0.001	0.012	0.004	0.006	0.261
Age	0.007	−0.002	0.001	0.005	0.026*	0.002	0.000	−0.000	0.001*	0.000	−0.000	0.000*	0.000	0.000	0.048
Gender (Male)	0.010	0.0712	0.029	0.346	0.349	0.013	−0.001	0.019*	0.029	−0.004	−0.011	0.019	0.000	−0.014	0.419
Admission (Inpatient)	5.395***	1.184***	3.445***	27.575***	13.489***	0.319	0.047**	0.003	0.234***	0.629***	1.052***	1.069	0.996***	0.068***	22.485***
Location (Nonprovince Center)	1.092	−0.1031	0.169	0.924	1.864	0.178	0.022	0.063	0.048	−0.039	0.032	0.041	0.003	0.004	2.728
Insurance (Insuree)	−0.405	0.153	0.083	0.294	0.256	−0.008	0.004	0.010	−0.020	0.010	0.007	0.018	−0.000	0.005	0.240
Autoimmune Diseases	1.543	0.507	0.719*	4.017***	4.407*	0.417	0.109*	0.047	0.044	0.210	−0.008	0.224***	0.006	0.017	4.694*
Cancers	1.117	−1.220	0.763*	−0.796	1.119	0.297	−0.072	−0.047	0.241*	0.919***	0.048	−0.085	0.003	0.271***	6.125*
Diabetes	0.244	−0.813*	0.294*	−1.150*	−0.704	0.055	−0.026	0.068**	0.017	−0.011	−0.020	0.015	−0.006	−0.023	−0.381
Cardiovascular Diseases	1.107*	1.045**	0.290*	1.804***	4.963***	0.247	0.038*	0.053**	0.094*	0.185*	0.036*	0.200***	0.000	−0.000	5.290***
Respiratory Diseases	−1.167*	1.588***	0.149	1.414*	6.138***	−0.057	0.052*	0.104***	0.120**	0.259	0.024	0.116**	−0.012*	−0.069**	5.798***
_cons	0.166	0.087	0.845***	−0.681	1.579	−0.113	−0.014	−0.018	−0.053	−0.012	0.008	−0.083*	−0.007	−0.008	1.861
	*F* = 50.66 *R* ^2^ = 0.360	*F* = 10.67 *R* ^2^ = 0.106	*F* = 216.95 *R* ^2^ = 0.707	*F* =1044.64 *R* ^2^ = 0.920	*F* = 118.56 *R* ^2^ = 0.569	*F* = 24.32 *R* ^2^ = 0.213	*F* = 5.88 *R* ^2^ = 0.061	*F* = 8.93 *R* ^2^ = 0.090	*F* = 23.92 *R* ^2^ = 0.210	*F* = 36.31 *R* ^2^ = 0.287	*F* = 918.74 *R* ^2^ = 0.910	*F* = 516.84 *R* ^2^ = 0.851	*F* = 10,238.17 *R* ^2^ = 0.991	*F* = 4.82 *R* ^2^ = 0.050	*F* = 150.19 *R* ^2^ = 0.625

* *p* Value is significant at the 0.10 level, *p* < 0.1

** *p* Value is significant at the 0.05 level, *p* < 0.05

*** *p* Value is significant at the 0.01 level, *p* < 0.01.

Compared to COVID‐19 outpatients, hospitalized patients use more diagnostic and therapeutic services, including consumables, surgery, nutrition counseling, blood gas tests, CT scans, radiology, rehabilitation services, medicine, laboratory services, visits, length of ICU hospitalization, and non‐ICU hospitalization.

Moreover, we evaluate how comorbidities affect COVID‐19 patients' use of diagnostic and treatment services concerning five other illnesses. COVID‐19 patients with autoimmune disorders utilize more diagnostic and treatment services, including consumables, echo, rehabilitation, medicines, laboratory tests, and visits. The use of diagnostic and treatment services for COVID‐19 patients with cancer increases, along with a significant rise in the use of consumables, surgeries, CT scans, radiology, and visits.

The utilization of diagnostic and treatment services significantly changes for COVID‐19 patients with diabetes. In this regard, visits and dialysis increase, while ICU hospitalization and laboratory services decrease.

There is a notable rise in the utilization of diagnostic and therapeutic measures for COVID‐19 patients who have underlying cardiovascular conditions. This encompasses a range of services, such as consumables, echo, blood gas tests, CT scans, radiology, dialysis, rehabilitation services, medications, laboratory services, visits, and hospital stays, both in intensive care and nonintensive care settings.

The overall utilization of diagnostic and therapeutic services for COVID‐19 patients with respiratory disorders significantly increases. This rise includes the use of consumables, echo, radiology, dialysis, rehabilitation services, medicines, laboratory services, and length of ICU hospitalization.

The second regression model analyzes how the number of doses affects the usage of medical services for COVID‐19 patients. The impact of contextual variables is also considered, and the results are presented in Table 3. In this model, administering two or three vaccination doses rather than one and withholding the vaccine reduces the duration of hospitalization in the ICU, non‐ICU stay, consumption of resources, CT scans, rehabilitation services, ultrasounds, medication, laboratory services, and visits.

**Table 3:** Variables affecting utilization of services based on the number of vaccine doses administered in Iran between 2021 and 2022.

	Visits and Medical Counseling	Laboratory	Medicine	Ultrasound	Rehabilitation	Dialysis	Radiology	CT scan	Arterial Blood Gas test	Echo	Nutrition Advice	Surgery	Medical Supplies	Length of non‐ICU stay	Length of ICU stay
1 Dose Administered (non‐vaccinated)	0.036	−0.056	0.237	0.021	−0.012	0.010	0.001	−0.014	0.011	0.001	0.005	0.025	1.230	0.106	−0.089
2 or 3 Doses Administered (non‐vaccinated)	−0.016	−0.065	−0.806	−0.015	−0.023	0.019	0.001	−0.035	−0.006	0.021	0.003	−0.008	−0.527	−0.461	−0.218
Age	0.001	0.005	0.036	0.002	0.000	−0.000	0.001	0.000	−0.000	0.000	0.000	0.000	0.064**	0.012	−0.001
Gender (Male)	0.030	0.346	0.357	0.013	−0.001	0.019*	0.029	−0.004	−0.011	0.019	0.000	−0.014	0.432	0.014	0.072
Patients (Inpatient)	3.445***	27.575***	13.501***	0.319***	0.047**	0.003	0.234***	0.629***	1.052***	1.068***	0.996***	0.069***	22.505***	5.401***	1.185***
Location (Non‐province Center)	0.163	0.923	1.743	0.174*	0.020	0.064*	0.048	−0.041	0.030	0.043	0.002	0.000	2.524	1.026	−0.118
Insurance (Insuree)	0.088	0.295	0.343	−0.005	0.005	0.010	−0.020	0.012	0.009	0.016	−0.000	0.008	0.386	−0.357	0.164
Autoimmune Diseases	0.723*	4.018***	4.483*	0.420**	0.110*	0.046	0.044	0.212	−0.006	0.223***	0.006	0.019	4.822*	1.585	0.516
Cancers	0.767*	−0.795	1.180	0.299*	−0.071	−0.047	0.241*	0.921***	0.049	−0.086	0.003	0.273***	6.227*	1.150	−1.213
Diabetes	0.299*	−1.149*	−0.613	0.058	−0.025	0.067**	0.017	−0.009	−0.019	0.013	−0.006	−0.020	−0.227	0.293	−0.802*
Cardiovascular Diseases	0.290*	1.804***	4.961***	0.247***	0.038*	0.053**	0.094	0.185*	0.036	0.200***	0.000	−0.000	5.286***	1.106*	1.045**
Respiratory Diseases	0.150	1.414*	6.160***	−0.056	0.052*	0.104***	0.120**	0.259**	0.025	0.116**	−0.012*	−0.068**	5.836***	−1.154*	1.591***
_cons	0.819***	−0.0685	1.067	−0.131	−0.019	−0.013	−0.053	−0.023	−0.000	−0.073	−0.008	−0.025	0.999	−0.112	0.023
	F = 198.75 R^2^ = 0.707	F = 956.62 R^2^ = 0.920	F = 109.12 R^2^ = 0.570	F = 22.35 R^2^ = 0.213	F = 5.43 R^2^ = 0.061	F = 8.22 R^2^ = 0.090	F = 21.90 R^2^ = 0.210	F = 33.27 R^2^ = 0.288	F = 843.04 R^2^ = 0.911	F = 473.86 R^2^ = 0.852	F = 9377.23 R^2^ = 0.991	F = 4.86 R^2^ = 0.055	F = 138.51 R^2^ = 0.627	F = 46.82 R^2^ = 0.362	F = 9.80 R^2^ = 0.106

* *P*‐value is significant at the 0.10 level, *p*‐value < 0.1

** *P*‐value is significant at the 0.05 level, *p*‐value < 0.05

*** *P*‐value is significant at the 0.01 level, *p*‐value < 0.01

In this model, men have a higher dialysis usage rate than women. Additionally, the consumption of diagnostic and treatment services increases with age. In comparison to outpatients with COVID‐19, hospitalized patients tend to require more frequent use of diagnostic and therapeutic services. This includes an elevated demand for consumables, surgery, nutrition counseling, echo, blood gas tests, CT scans, radiology, rehabilitation services, ultrasounds, medicines, laboratory services, visits, and a longer hospitalization period in both the ICU and non‐ICU setting.

Furthermore, we assess the impact of comorbidities on the utilization of diagnostic and treatment services for COVID‐19 patients, compared to five other illnesses. The overall use of diagnostic and treatment services for COVID‐19 patients with autoimmune disorders increases, with a significant rise in consumables, echoes, rehabilitation services, ultrasounds, medicines, laboratory services, and visits.

COVID‐19 patients with cancer experience prolonged hospital stays, increased demand for nutritional counseling and blood gas tests, heightened medication usage, and a significant surge in the utilization of consumables, surgery, CT scans, radiology, ultrasounds, and doctor visits.

The use of diagnostic and therapeutic services increases for COVID‐19 patients with diabetes, including ultrasounds, radiology, echocardiography, and non‐ICU hospitalization length. Additionally, there is a significant increase in visits and dialysis services.

The use of diagnostic and therapeutic services for COVID‐19 patients with cardiac disorders leads to a significant increase in hospitalization, consumables, and medical services. Also, COVID‐19 patients with respiratory disorders require increased use of diagnostic and therapeutic services, including ICU hospitalization, consumables, echo, CT scans, radiology, dialysis, rehabilitation services, medicines, and laboratory tests.

Overall, regression estimates show that vaccination decreases medical utilization in COVID‐19 patients by controlling for confounding factors, such as age and comorbidities.

## 4 DISCUSSION

The study aimed to investigate how vaccination and the number of doses injected before COVID‐19 infection impacted healthcare utilization in confirmed cases. Regression estimates revealed that vaccination reduced healthcare service utilization for COVID‐19 patients, after controlling for confounding variables. However, univariate analysis tests showed that vaccination increased mean healthcare service utilization for COVID‐19 patients in general. According to the study, vaccines and booster doses play a crucial role in reducing medicine, medical supplies, and the length of hospitalization in both ICU and non‐ICU departments.

Vilches et al. found that patients who received the Pfizer‐BioNTech and Moderna vaccines experienced a significant decrease of 27.3% and 27.0%, respectively, in hospitalization rates in Ontario, Canada.^33^ López et al. conducted a study indicating that the vaccination of Catalonia could potentially result in an 18% reduction in ICU hospitalizations, a 16% reduction in hospital admissions, a 5% decrease in PCR testing utilization, and a 1% decrease in RAT testing utilization.^34^ Also, Hall et al. state that vaccination not only lessens the occurrence of COVID‐19 symptoms but also diminishes the spread of infection, In England.^35^ Moghadas et al. found that giving priority to adults aged 65 and above during vaccination could lower the overall attack rate from 9% to 4.6%. It could also decrease the death rate to 69.3%, the non‐ICU hospitalization rate to 63.5%, and the ICU hospitalization rate to 65.6%.^36^

Regarding the use of COVID‐19 health services, factors including type of vaccine as well as variants of the virus play a significant role that will be regarded in the following. According to Munir et al.'s research, individuals who received the messenger RNA (mRNA) vaccine (Moderna) reported significantly higher postvaccination side effects (85%) compared to those who received the inactivated virus vaccine (Sinovac) (21%).^37^ McLean et al. found that current vaccines are effective against the Alpha variant but not as effective against the Beta and Delta variants for causing symptoms. Boosters help restore protection but may decrease over time.^38^

Patients with comorbidity diseases show increased utilization of diagnostic and therapeutic services when infected with COVID‐19. The utilization of services also varies significantly depending on the type of comorbidity, with autoimmune, cardiac, and respiratory disorders showing increased utilization in several areas. In terms of medicines, visits, consumables, laboratory tests, radiology, and length of ICU hospitalization, the utilization of diagnostic and therapeutic services in COVID‐19 patients with autoimmune, cardiac, and respiratory disorders increases significantly. In COVID‐19 patients with diabetes, the length of stay in the ICU and the use of laboratory services both significantly decrease while visits and dialysis both significantly increase in the same group. The utilization of consumables, surgery, CT scans, radiology, ultrasounds, and visits increases significantly for COVID‐19 patients with cancer.

A study in India found that 21.4% of those who died from COVID‐19 had underlying health conditions. After receiving both vaccine doses, the mortality rate for patients with comorbidities dropped to 0.6%.^39^ Cancer patients with weakened immune systems may need shorter intervals between COVID‐19 vaccine doses. Vaccination programs should prioritize this population for booster doses.^40^ The adjusted odds ratios of COVID‐19 patients with comorbidities such as cardiovascular disorders, immunosuppressive diseases, and diabetes, respectively, are 1.79, 1.65, and 1.41, according to research by De Rosa et al. in Italy.^41^ People with weak immune systems may not get the same protection from COVID‐19 vaccination as healthy people, leading to a higher risk of severe side effects, intensive care requirement, and higher mortality rate.^42^

The effectiveness of vaccination programs is hindered by various factors, in particular, in Iran, including the lack of timely reminder doses, international sanctions, cultural and social concerns have led to a lack of public acceptance of vaccination, delays in establishing infrastructure for widespread vaccine distribution,^43^ global vaccine supply constraints,^44^ prioritization of high‐risk groups for vaccination,^43^ and varying efficacy of different types of vaccines against new variants like the Omicron.^45^ The widespread use of inactivated virus vaccines like Sinopharm may not be as effective as viral vector or mRNA vaccines against new variants.^46^ Furthermore, mobility restrictions implemented in response to COVID‐19 have led to a decline in medical service utilization, exacerbating health outcomes, particularly maternal and child mortality rates.^47^ In this regard, overall healthcare utilization decreased by 37%, with significant declines in visits (42%), admissions (28%), diagnostics (31%), and treatments (30%), and reductions varied with illness severity, with milder cases showing more significant decreases.^48^ In this regard, a study in Ethiopia found managing COVID‐19 patients was costly, especially for the elderly and those with comorbidities, which governments should prioritize vaccinating these high‐risk groups to reduce the financial burden on health facilities.^49^ Also, considering how COVID‐19 spreads and related climate factors are vital for effective public health measures. Officials can use the virus's seasonal pattern and low humidity and high temperatures during summer months to reduce transmission rates.^50^

COVID‐19 variants and economic challenges have made vaccination campaigns less effective and strained resources for patients. Addressing these challenges and navigating future health crises demand a multifaceted approach that encompasses vaccine delivery, supply chain optimization, vaccine development, tailored guidelines, behavioral modifications, strategic lockdowns, improving vaccination acceptance and targeted vaccination campaigns, all underpinned by strong governance, adequate public investments, and supportive sociocultural factors.^30^, ^51^, ^52^, ^53^, ^54^, ^55^ With regard to crisis management, adopting strategies and new technology that involve minimal strict containment restrictions are more efficient.^53^ For instance, a Canadian study found that a remote care program known as COVIDEO, a program offered virtual consultations for COVID‐19 patients, reduced emergency department visits, hospitalizations, or deaths and facilitated direct‐to‐ward hospitalizations.^56^

## 5 CONCLUSIONS

We searched and prepared the study without finding any other studies in Iran that were comparable. However, this study has some limitations that need to be addressed. These include registration errors, errors in the use of provided services and clinical information from the integrated health system, and the lack of available data on the vaccine type administered to patients as well as infecting variants of the virus for evaluating vaccine effectiveness. Therefore, further research is needed using advanced statistical methods to reach a definite conclusion due to ambiguous results, study limitations, and contextual factors not considered.

Full vaccination, along with contextual factors, plays a critical role in the healthcare service utilization of COVID‐19 patients. By expanding universal vaccination, decision‐makers can suppress rising utilization and expenditures of health services induced by COVID‐19. Future pandemics require a comprehensive approach and significant investments in public health infrastructure, global cooperation, and efficient vaccination plans for high‐risk groups.

## AUTHOR CONTRIBUTIONS

**Erfan Kharazmi**: Supervision; validation. **Mohsen Bayati**: Data curation; formal analysis; methodology; software. **Ali Majidpour Azad Shirazi**: Conceptualization; data curation; investigation; project administration; resources; writing—original draft; writing—review and editing.

## CONFLICT OF INTEREST STATEMENT

The authors declare no conflict of interest.

## ETHICS STATEMENT

The project was found to be by the ethical principles and the national norms and standards for conducting medical research. The study protocol was approved by the ethics committee of Shiraz University of Medical Sciences under code IR.SUMS.NUMIMG.REC.1401.037. We used aggregate utilization data so informed consent was not required. Approval Date: 2022‐05‐14; Approval ID: IR.SUMS.NUMIMG.REC.1401.037.

## TRANSPARENCY STATEMENT

The lead author Ali Majidpour Azad Shirazi affirms that this manuscript is an honest, accurate, and transparent account of the study being reported; that no important aspects of the study have been omitted; and that any discrepancies from the study as planned (and, if relevant, registered) have been explained.

## Supporting information

Supporting information.

## Data Availability

The datasets gathered and analyzed during the current study are available from the corresponding author upon reasonable request. The corresponding author has full access to all of the data in this study and takes complete responsibility for the integrity of the data and the accuracy of the data analysis.
