# Supplementary material for: Vaccination and its impact on healthcare utilization in two groups of vaccinated and unvaccinated patients with COVID‐19: A cross‐sectional study in Iran between 2021 and 2022
Source: Health Sci Rep. 2024 Feb 23;7(2):e1914. doi: 10.1002/hsr2.1914 (PMC10885182; doi:10.1002/hsr2.1914)
Supplement: Supplementary file 1 — Supporting information. [file HSR2-7-e1914-s001.docx]

| Table S1: The mean difference of service utilization variables based on vaccination in Iran between 2021 and 2022 | | | | | | |
| --- | --- | --- | --- | --- | --- | --- |
| Utilization of services | **Shapiro-Wilk W test for normality test** | | **Vaccination** | | **Mean difference test** | |
|  | **W** | **P-Value** | **Non-Vaccinated** | **Vaccinated** | **t \ z** | **P-Value** |
| Visits** | 0.944 | 0.000 | Mean=1.966  (1.750) | Mean=2.391  (2.220) | z=-2.406 | P= 0.016 |
| Length of non-ICU stay** | 0.694 | 0.000 | Mean=1.696  (4.140) | Mean=2.157  (4.927) | z=-2.188 | P= 0.028 |
| Length of ICU stay | 0.653 | 0.000 | Mean=0.508  (2.833) | Mean=0.609  (2.996) | z=-1.584 | P= 0.113 |
| Laboratory** | 0.957 | 0.000 | Mean= 7.585  (12.872) | Mean=10.528  (14.507) | z=-2.477 | P= 0.013 |
| Medicine*** | 0.858 | 0.000 | Mean=7.237  (9.309) | Mean= 9.473  (11.837) | z=-2.664 | P= 0.007 |
| Ultrasound | 0.855 | 0.000 | Mean=0.099  (0.335) | Mean=0.190  (0.613) | z=-1.226 | P= 0.220 |
| Rehabilitation | 0.846 | 0.000 | Mean=0.027  (0.195) | Mean= 0.026  (0.183) | z= -0.085 | P= 0.931 |
| Dialysis** | 0.832 | 0.000 | Mean=0  (0) | Mean= 0.029  (0.195) | z=-2.123 | P= 0.033 |
| Radiology** | 0.929 | 0.000 | Mean=0.066  (0.309) | Mean= 0.126  (0.381) | z=-2.444 | P= 0.014 |
| CT scans | 0.857 | 0.000 | Mean= 0.204  (0.621) | Mean= 0.295  (0.772) | z=-1.528 | P= 0.126 |
| Arterial blood gas test** | 0.971 | 0.000 | Mean= 0.287  (0.489) | Mean= 0.390  (0.540) | z=-2.425 | P= 0.015 |
| Echo*** | 0.976 | 0.000 | Mean=0.292  (0.513) | Mean= 0.451  (0.653) | z=-2.812 | P= 0.004 |
| Nutrition*** Advice | 0.998 | 0.520 | Mean= 0.265  (0.442) | Mean= 0.365  (0.481) | t=-2.5607 | P= 0.010 |
| Surgery | 0.719 | 0.000 | Mean= 0.011  (0.148) | Mean=0.021  (0.196) | z=-1.066 | P= 0.286 |
| Medical supplies** | 0.748 | 0.000 | Mean= 10.966  (12.144) | Mean= 14.978  (17.438) | z=-2.170 | P= 0.030 |
| (…) Indicates Standard Deviation  *** P-value is significant at the 0.01 level, p-value <0.01  ** P-value is significant at the 0.05 level, p-value <0.05  * P-value is significant at the 0.10 level, p-value <0.1 | | | | | | |

| Table S2: The mean difference of service utilization variables based on the number of vaccine doses administered to the participant in Iran between 2021 and 2022 | | | | | | |
| --- | --- | --- | --- | --- | --- | --- |
| Utilization of services | **P-Value Significant** | | **Vaccination** | | |  |
|  |  |  | 2 or 3 doses administered | 1 dose administered | Non-Vaccinated |  |
| Visits and medical counseling *** | P= 0.000 | F= 15.92 | Mean= 2.658  (2.337) | Mean= 1.861 (1.860) | Mean=1.966  (1.750) |  |
| Length of non-ICU stay ** | P= 0.019 | F= 3.96 | Mean= 2.460  (5.077) | Mean=1.554 (4.565) | Mean=1.696 (4.140) |  |
| Length of ICU stay | P= 0.358 | F= 1.03 | Mean= 0.710  (3.098) | Mean= 0.408 (2.778) | Mean=0.508 (2.833) |  |
| Laboratory *** | P= 0.000 | F= 20.50 | Mean=12.563  (15.081) | Mean= 6.481 (12.357) | Mean=7.585 (12.872) |  |
| Medicine *** | P= 0.000 | F= 12.49 | Mean= 10.702  (12.408) | Mean=7.029 (10.199) | Mean=7.237 (9.309) |  |
| Ultrasound *** | P= 0.001 | F= 6.40 | Mean= 0.233  (0.663) | Mean= 0.105 (0.491) | Mean= 0.099 (0.335) |  |
| Rehabilitation | P= 0.405 | F= 0.90 | Mean= 0.033  (0.207) | Mean= 0.014 (0.120) | Mean= 0.027 0.195 |  |
| Dialysis ** | P= 0.014 | F= 4.26 | Mean= 0.038  (0.219) | Mean= 0.010 (0.134) | Mean= 0  (0) |  |
| Radiology *** | P= 0.000 | F= 8.47 | Mean= 0.159  (0.426) | Mean= 0.062 (0.256) | Mean= 0.066 (0.309) |  |
| CT scans *** | P= 0.001 | F= 6.83 | Mean= 0.357  (0.841) | Mean= 0.171 (0.595) | Mean= 0.204 0.621 |  |
| Arterial blood gas test *** | P= 0.000 | F= 16.56 | Mean= 0.458  (0.544) | Mean= 0.255 (0.506) | Mean= 0.287 (0.489) |  |
| Echo *** | P= 0.000 | F= 26.52 | Mean= 0.552  (0.700) | Mean= 0.251 (0.490) | Mean= 0.292 (0.513) |  |
| Nutrition Advice *** | P= 0.000 | F= 20.62 | Mean= 0.229  (0.421) | Mean= 0.229 (0.421) | Mean= 0.265 (0.442) |  |
| Surgery | P= 0.394 | F= 0.93 | Mean= 0.016  (0.165) | Mean= 0.032 (0.247) | Mean= 0.011 (0.148) |  |
| Medical supplies *** | P= 0.000 | F= 14.29 | Mean= 16.790  (17.723) | Mean= 11.372 (16.297) | Mean= 10.966 (12.143) |  |
| (…) Indicates Standard Deviation  *** P-value is significant at the 0.01 level, p-value <0.01  ** P-value is significant at the 0.05 level, p-value <0.05  * P-value is significant at the 0.10 level, p-value <0.1  Graphs in the table depict the pattern of changes in the utilization of diagnostic and treatment services | | | | | | |
